# Opportunities and challenges for the development of polymer-based biomaterials and medical devices

**DOI:** 10.1093/rb/rbw008

**Published:** 2016-03-08

**Authors:** Jinghua Yin, Shifang Luan

**Affiliations:** ^1^WEGO Holding Company Limited, Weihai 264210, People's Republic of China;; ^2^State Key Laboratory of Polymer Physics and Chemistry, Changchun Institute of Applied Chemistry, Chinese Academy of Sciences, Changchun 130022, People's Republic of China

**Keywords:** polymer-based biomaterials, antibacterial, plasticizer, ethylene oxide sterilization, anti-irradiation aging

## Abstract

Biomaterials and medical devices are broadly used in the diagnosis, treatment, repair, replacement or enhancing functions of human tissues or organs. Although the living conditions of human beings have been steadily improved in most parts of the world, the incidence of major human’s diseases is still rapidly growing mainly because of the growth and aging of population. The compound annual growth rate of biomaterials and medical devices is projected to maintain around 10% in the next 10 years; and the global market sale of biomaterials and medical devices is estimated to reach $400 billion in 2020. In particular, the annual consumption of polymeric biomaterials is tremendous, more than 8000 kilotons. The compound annual growth rate of polymeric biomaterials and medical devices will be up to 15–30%. As a result, it is critical to address some widespread concerns that are associated with the biosafety of the polymer-based biomaterials and medical devices. Our group has been actively worked in this direction for the past two decades. In this review, some key research results will be highlighted.

## Opportunities for the development of polymer-based biomaterials and medical devices

Biomaterials and medical devices industry have been rapidly developed in the past 10 years, thanks to the advances in science and technology and the tremendous clinical demand. The compound annual growth rate (CAGR) of biomaterials and medical devices is up to 15% in the 21st century. The global market sale of biomaterials and medical devices was about $203.5 billion in 2013. Although the living conditions of human beings have been steadily improved in most parts of the world, the incidence of major human’s diseases is still rapidly growing, which is mainly attributed to population growth and aging. For example, the global cancer cases are projected to raise from 14 million in 2012–19 million in 2025 and 24 million in 2035, respectively. According to the International Diabetes Federation, the number of population worldwide with diabetes was around 382 million in 2013. More strikingly, this number is estimated to reach 522 million in 2035. As another example, according to World Health Organization, cardiovascular disease is the leading cause of death worldwide, accounting for 29% of the global mortality. The number of deaths will increase from about 17.5 million in 2013–25 million in 2020 and 36 million in 2035, respectively.

The CAGR of biomaterials and medical devices is projected to maintain ∼10% growth in the next 10 years. The global market sales of biomaterials and medical devices will reach $400 billion in 2020. In particular, the market sale in China was $15.1 billion in 2013, which accounts for less than 8% of the worldwide sale; however, the number is estimated to rapidly grow to $71.5 billion in 2020 and $302.9 billion in 2035, respectively.

Among various biomaterials, the annual consumption of polymer-based biomaterials is tremendous, more than 8000 kilotons [1]. However, only a few dozen species of polymeric materials, such as polyolefin, polyvinyl chloride (PVC), medical engineering plastics and so forth, have been put into large-scale application to fabricate syringe, drug and blood storage and transfusion consumable, catheter, orthopedic device and so forth. Currently, the annual consumption of polyolefin, PVC and medical engineering plastics reaches more than 1500, 1000 and 200 kilotons, respectively. It is predicted that the polymer-based biomaterial market will grow at a high rate of 15–30% annually in the 21st century.

## Challenges for the development of polymer-based biomaterials and medical devices

Recently, the safety of polymer-based biomaterials and medical devices in service, e.g. device-associated bacterial infections, the hazards of plasticized PVC biomaterials and the defects of ethylene oxide (EO) sterilization, has caused widespread concerns. To address this, our group has been actively worked in this direction since 2000. In specific, two key scientific issues, i.e. the relationships between the modification methods of polymers and their performances, and the interactions between the surfaces of polymers and blood, cell and microorganism, have been investigated. In this article, some key progresses are highlighted.

### Bacteria-repellent materials and antibacterial polymers

Despite of sterilization and aseptic procedures, bacterial infection remains a major impediment to the usage of medical devices (Table 1) [2]. In the USA, indwelling devices were responsible for over half of all nosocomial infections, with an estimate of 1 million cases per year [3]. Since 2001, 2.6 million orthopedic implants were inserted into humans per year, among which ∼4.3% became infected [4]. Estimates of the direct medical costs associated with microorganism infections exceed $3 billion annually. It is expected the number of device-associated infections will continue to grow as more patients receive biomedical implants [3]. A potent approach to combat the device-associated infections is to develop novel antimicrobial materials (surfaces) via introducing antibiotics and biocides [5]. However, antibiotics and biocides are generally connected with the risk of high cytotoxicity or antibiotic resistance, which has already raised great concerns because of their potential threat to human and environmental health [6]. An alternative bacteria-repellent approach that renders the biomaterials resistant to bacterial attachment has been developed through constructing antifouling coatings [7]. Although this passive approach has good biocompatibility, it is not capable to kill the adherent bacteria, making it hard to avoid the ultimate infection [8].

**Table 1.** Incidence of biomaterial-associated infection for different implants and devices [2]

Tissue implant site	Implant or device	Infection incidence over lifetime (%)
Urinary tract	Catheter	33 (per week)
Percutaneous	Central venous catheter	2–10
	Temporary pacemaker	4
	Short indwelling catheter	0–3
	Peritoneal dialysis catheter	3–5
	Fixation pin or screw	5–10
	Suture	1–5
	Voice prosthesis	25 (per month)
	Dental implant	5–10
Subcutaneous	Cardiac pacemaker	1–7
	Penile prosthesis	2–5
Soft tissue	Mammary prosthesis	1–7
	Abdominal wall patch	1–16
	Intraocular lens	0.1
Eye	Contact lens	0.1–0.5
Circulatory system	Prosthetic heart valve	1–3
	Vascular graft	1.5
Bone	Prosthetic hip	2–4
	Prosthetic knee	3–4
	Tibial nail	1–7

Incidence data are given over the entire implant or device lifetime, unless stated otherwise

**Figure 1.**
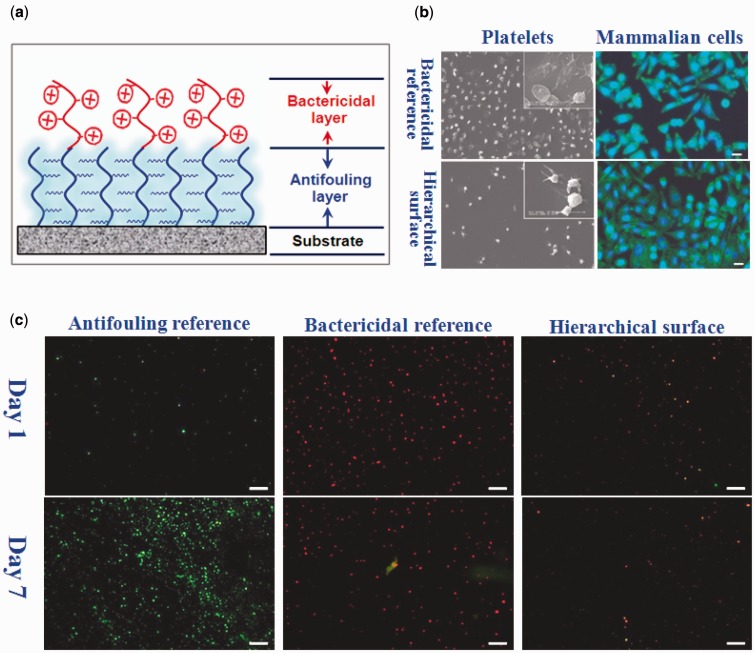
(**a**) Hierarchical surface consisting of a PEG antifouling bottom layer and a QAC bactericidal top layer, (**b**) the adhesion of platelets and mammalian cells on the samples, (**c**) representative confocal laser scanning microscopy images of the *Staphylococcus aureus* adhered on the samples. PBS suspension of the bacteria (10^6^ cells ml^−1^) was dropped onto the surfaces of the samples. After incubating for 1 day, the samples were washed with PBS to remove the non-adherent bacteria, followed by dropping fresh culture medium onto the surfaces every 24 h for 7 days

**Figure 2.**
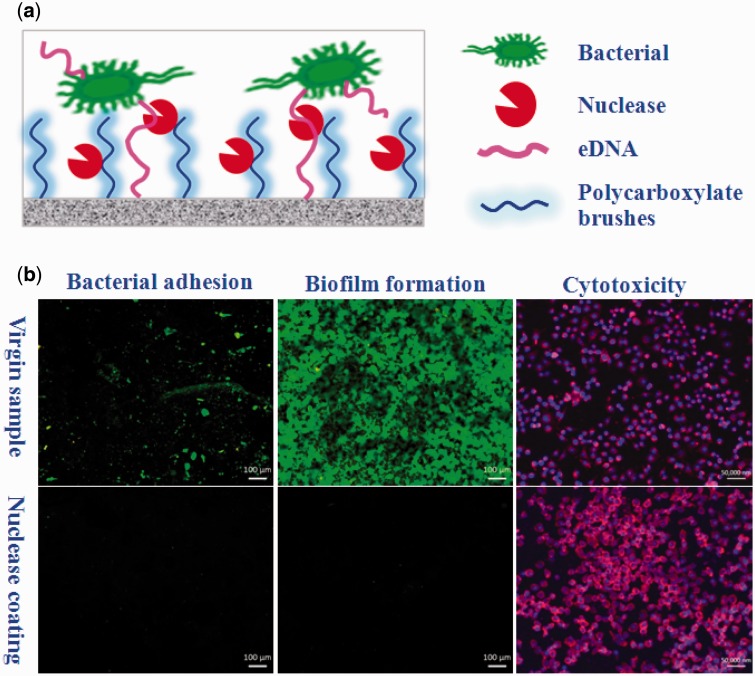
(**a**) Illustration of the DNase coating to cleave DNA and (**b**) bacterial adhesion, biofilm formation and cytotoxicity

**Figure 3.**
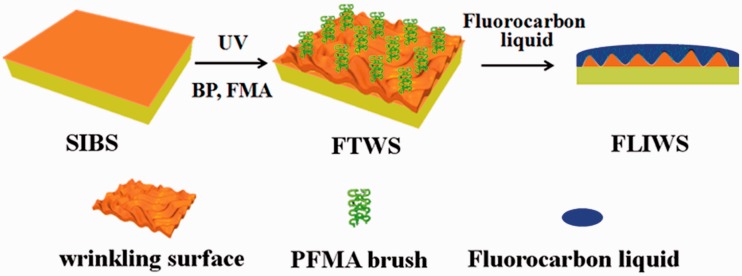
Illustration of the fluorocarbon liquid-infused wrinkling slippery surface to repel bacteria

On the basis of the bacteria-repellent mechanism, our group has developed a series of infection-resistant surfaces:

(1) Hierarchical polymer brushes comprising bacteria-repellent and bactericidal capabilities (Yan *et al.*, unpublished results). A surface-initiated photoiniferter-mediated polymerization strategy is proposed to construct a robust antibacterial surface that consists of a poly (ethylene glycol) (PEG) antifouling bottom layer and a quaternary ammonium compound (QAC) bactericidal top layer (Fig. 1). In this hierarchical architecture, the PEG layer serves as an effective antifouling background to suppress bacterial attachment, and the QAC layer offers a killing response to the opportunistic settlement of microbes. Ultimately, an excellent long-term antibacterial surface with integrated bacteria-repelling and bactericidal capability has been constructed, compared with the PEG antifouling reference and the QAC bactericidal reference. In addition, the hierarchical samples had a low toxicity towards mammalian cells, likely due to the PEG background layer and the low density of QAC top layer. The hierarchical polymer brush system provides the basis for the development of the long-term infection-resistant and biocompatible surfaces. Similar approaches can also be applied with a variety of monomeric building blocks.

(2) Deoxyribonuclease (DNase) coating to prevent bacterial adhesion and biofilm formation [9]. A DNase coating was constructed on the surface of a polycarboxylate-modified polymer substrate under mild conditions (Fig. 2). It is known that DNase can attack and cleave extracellular deoxyribonucleic acid (eDNA), which is critical for bacterial adhesion and biofilm formation [10, 11]; therefore, the DNase coating is capable to effectively prevent bacterial infections without causing biocide resistance. The as-prepared DNase coating is considered as a promising approach to inhibit bacterial infection while preserving tissue-cell integration on polymeric biomaterials.

(3) An infection-resistant slippery surface through infusing fluorocarbon-tethered wrinkling surface with fluorocarbon liquid [12]. Inspired by the liquid-infused porous slippery surface [13], a fluorocarbon-tethered wrinkling surface was facilely prepared by combining photo-graft polymerization with osmotically driven wrinkling, followed by infusing with perfluorooctyl methacrylate liquid lubricant to obtain a fluorocarbon liquid-infused wrinkling surface (Fig. 3) [12]. Fluorocarbon liquid-infused wrinkling surface is characterized by the following features. The affinity between the perfluorocarbon liquid and the fluorocarbon-tethered surface is much higher than that between the ambient fluid and the surface. The perfluorocarbon lubricating fluid is locked in place to form a stable, defect-free, self-healing and inert slippery surface, because the roughness of the surface is greatly increased by the osmotically driven wrinkling. The slippery surface repels various liquids, thus can resist infection and thrombus formation.

### Plasticizer-free polymers for biomedical applications

Biomedical soft PVC is used in the production of a wide range of disposable medical devices including blood bag, infusion device, respiratory mask, peritoneal dialysis bag and catheter. About 2.4 million ton of PVC is consumed per year which accounts for 28% of the total polymer-based biomaterials [14]. In particular, diethyl hexyl phthalate (DEHP) is the most commonly used plasticizers in biomedical PVC. In general, DEHP is not chemically bound to PVC backbone and may leach from materials upon contacting with blood, drugs and intravenous injection fluids in service (Table 2) [15]. Available toxicological testing in animals and *in vitro* tests provide evidences for the association of DEHP and its metabolites with a wide range of adverse effects in multiple organ systems such as liver, reproductive tract (testes, ovaries and secondary sex organs), kidney, lung and heart [15].

**Table 2.** Human exposure to DEHP following treatment with PVC medical devices [16]

Treatment	Total exposure (mg) per patient	Time period	Body weight (mg/kg)
Hemodialysis	0.5–360	Dialysis session	0.01–7.2
Blood transfusion	14–600	Treatment	0.2–8.0
Extracorporeal oxygenation	–	Treatment period	42.0–140.0
Cardiopulmonary bypass	2.3–168	Treatment day	0.03–2.4
Artificial ventilation	0.001–4.2	Hour	–
Exchange transfusions	–	Treatment	0.8–4.2

**Figure 4.**
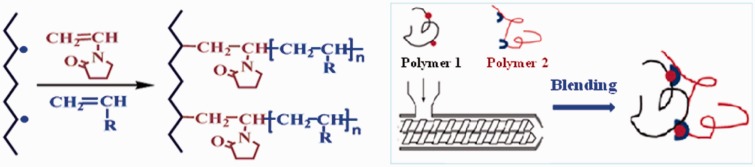
The chemical modification (left) and blending (right) principles

**Figure 5.**
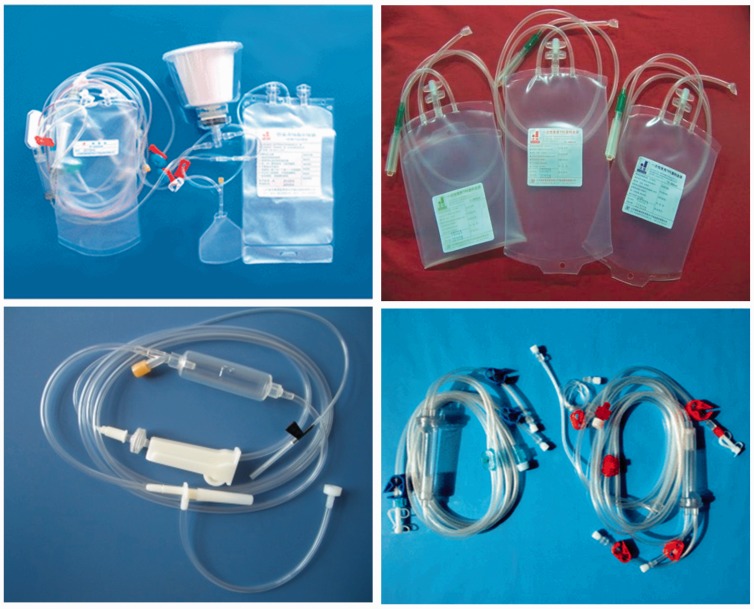
Representative TPE medical devices

Because of the potential hazard of soft PVC, various non-PVC alternatives have been investigated and developed in our group since 2000. In one study, styrene thermoplastic elastomers (TPE) were first chemically modified with acrylate through vinyl pyrrolidone intermediary bridge, followed by reactive blending with polypropylene to obtain TPE alloy (Fig. 4) [16–19]. According to Chinese National Standard GB 15593-1995 and GB 8368–2005, most of the biomedical properties of the as-prepared TPE alloys and associated medical devices were much better than those of the soft PVC ones (Table 3). Platelet storage period in TPE blood bag was up to 3–5 days, in stark contrast to 1–3 days in soft PVC one (Table 4). The TPE biomaterials and medical devices have been put into large-scale industrial production in WEGO HOLDING CO., LIMITED (China). Representative pictures of the TPS medical devices are shown in Fig. 5.

**Table 3.** Chemical and biological performances of TPE infusion device

Item	TPE device	PVC device
Reducing substance (ml/l)	0.1	1.2
pH change	0.2	1.0
Heavy metals (ppm)	Undetected	1.0
UV absorbance	0.001	0.03
Alcohol extraction (µg/ml)	Undetected	170
Cell toxicity	1 class	2 class
Intracutaneous stimulation	1 class	2 class
Hemolysis ratio (%)	0.2	2

**Table 4.** Storage performances of platelets in TPE blood bag

Item	TPE device	PVC device	*t* value
Number (×10^9^/l)	820 ± 140	688 ± 159	2.9
pH value	6.93 ± 0.08	5.59 ± 0.04	45.1
Platelet aggregation (%)	10.0 ± 8.8	0.4 ± 0.8	2.9
Hypotonic shock (%)	75.1 ± 4.2	0.3 ± 0.7	7.3
CD_62_P positive expression (%)	32.0 ± 4.6	83.1 ± 2.9	−18.8
Lactic acid (mmol/l)	14.84 ± 1.85	23.68 ± 8.14	−2.7
Glucose (mmol/l)	15.8 ± 1.0	5.8 ± 0.4	32.5

### Radiation-resistant polymer-based biomaterials

**Figure 6.**
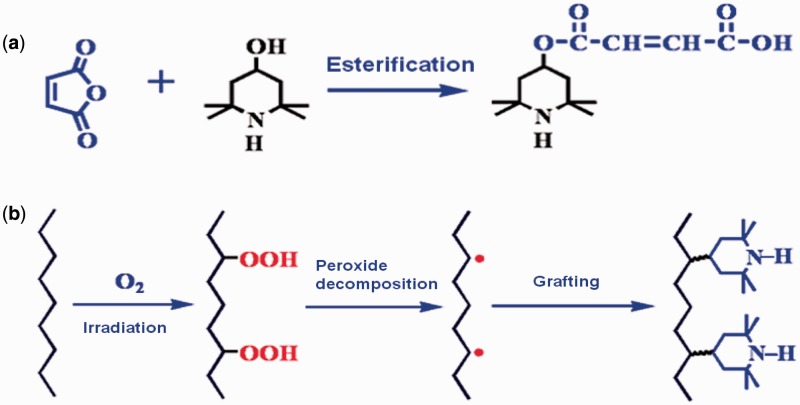
(**a**) Synthesis of reactive anti-irradiation agent and (**b**) reactive extrusion graft polymerization of anti-irradiation agent

**Figure 7.**
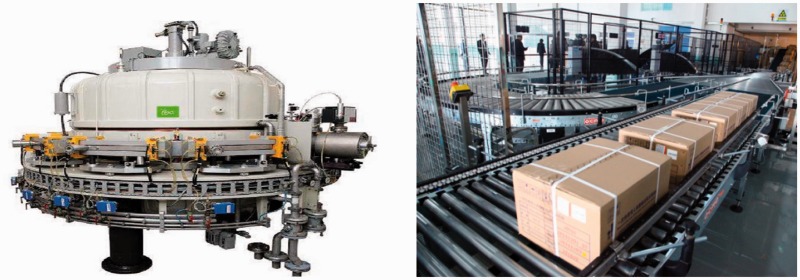
High-power cyclotron resonant cavity (left) and irradiation sterilization center in WEGO HOLDING CO., LIMITED (right)

Medical sterilization has become increasingly complex because of the need to prevent patient exposure to infections caused by instruments and devices [20]. Significant institutional costs related to nosocomial infections and mortality/morbidity concerns arise from the inadequate sterilization of medical devices [21]. Currently, the widely used industrial sterilization technologies of medical devices are steam, EO and irradiation. Each technology has its undeniable advantages over the other technologies. Notably, the defects of EO sterilization have aroused widespread concerns, mostly related to potential hazards of carcinogenic and mutagenic EO to patients, staffs and the environment, as well as risks associated with handling a flammable gas [22]. An investigation of symptoms in EO sterilization workers in hospital has confirmed that the daily sterilization work with EO can induce acute or chronic symptoms in EO sterilization workers [23]. Radiation sterilization is proven to be an effective method to kill the microorganism on material through electromagnetic radiation. Because of its outstanding effect in eliminating toxic and problematic residues, radiation sterilization of disposable medical devices has captured a large and still rapidly growing segment of the market in many industrialized countries [24]. However, irradiation sterilization usually causes the chain cracking of polymers and the creation of macro-radicals, resulting in the stiffening, discoloration and decreasing of mechanical properties of polymer-based materials [25–28]. Anti-irradiation agents are commonly mixed or added into bulk polymers to obtain irradiation-resistant capability [29]. Although this strategy is simple and efficient, the anti-irradiation agent may leach from materials, threating the long-term anti-irradiation performance. To solve this issue, we invent a pre-irradiation graft technology that produce macromolecular peroxide R–O–O–R or R–O–O–H to initiate the efficient graft polymerization of the double-bond-containing anti-irradiation agents in twin-screw extrusion (Fig. 6) [30, 31]. It is shown that, after being subjected to 25 kGy radiation dose and stored for 3 months, the tensile strength, yellowness index and haze of the as-prepared radiation-resistant PP material are obviously superior to the reference sample (Table 5). Four-category radiation-resistant polymeric biomaterials have been put into large-scale industrial production, and an irradiation sterilization center with annual capacity of 150 000 m^3^ has been built in WEGO HOLDING CO., LIMITED (China) (Fig. 7).

**Table 5.** Performances of radiation-resistant polymers^a^

Material	Tensile strength (MPa)	Flexural modulus (MPa)	Yellowness index	Haze (%)
PP	8.2	837	16	21.2
Anti-irradiation PP	27.2	971	8	1.8

^a^Twenty-five-kilogray radiation dose, stored for 3 months.

## References

[1] Vert M. Polymeric biomaterials: strategies of the past vs. strategies of the future. Prog Polym Sci 2007;32:755–61.

[2] Busscher HJ van der Mei HC Subbiahdoss G Biomaterial-associated infection: locating the finish line in the race for the surface. Sci Trans Med 2012;4:153rv10. 10.1126/scitranslmed.3004528 23019658

[3] Hetrick EM Schoenfisch MH. Reducing implant-related infections: active release strategies. Chem Soc Rev 2006;35:780–9. 16936926 10.1039/b515219b

[4] Darouiche RO. Treatment of infections associated with surgical implants. New Engl J Med 2004;350:1422–9. 15070792 10.1056/NEJMra035415

[5] Campoccia D Montanaro L Arciola CR. A review of the biomaterials technologies for infection-resistant surfaces. Biomaterials 2013;34:8533–54. 23953781 10.1016/j.biomaterials.2013.07.089

[6] Ferreira L Zumbuehl A. Non-leaching surfaces capable of killing microorganisms on contact. J Mater Chem 2009;19:7796–806.

[7] Banerjee I Pangule RC Kane RS. Antifouling coatings: recent developments in the design of surfaces that prevent fouling by proteins, bacteria, and marine organisms. Adv Mater 2011;23:690–718. 20886559 10.1002/adma.201001215

[8] Tuson HH Weibel DB. Bacteria-surface interactions. Soft Matter 2013;9:4368–80. 23930134 10.1039/C3SM27705D PMC3733390

[9] Yuan SS Zhao J Luan SF Nuclease-functionalized poly(styrene-b-isobutylene-b-styrene) surface with anti-infection and tissue integration bifunctions. ACS Appl Mater Interfaces 2014;6:18078–86. 25253647 10.1021/am504955g

[10] Whitchurch CB Tolker-Nielsen T Ragas PC Extracellular DNA required for bacterial biofilm formation. Science 2002;295:1487. 11859186 10.1126/science.295.5559.1487

[11] Ghaz-Jahanian MA Khodaparastan F Berenjian A Influence of small RNAs on biofilm formation process in bacteria. Mol Biotechnol 2013;55:288–97. 24062263 10.1007/s12033-013-9700-6

[12] Yuan SS Luan SF Yan SJ Facile fabrication of lubricant-infused wrinkling surface for preventing thrombus formation and infection. ACS Appl Mater Interfaces 2015;7:19466–73. 26268298 10.1021/acsami.5b05865

[13] Wong TS Kang SH Tang SKY Bioinspired self-repairing slippery surfaces with pressure-stable omniphobicity. Nature 2011;477:443–7. 21938066 10.1038/nature10447

[14] FDA Safety Assessment of Di(2-ethylhexyl)phthalate (DEHP) Released from PVC Medical Devices. http://wwwfdagov/downloads/MedicalDevices/DeviceRegulationandGuidance/GuidanceDocuments/UCM080457pdf. 21 February 2008, date last accessed.

[15] Tickner JA Schettler T Guidotti T Health risks posed by use of di-2-ethylhexyl phthalate (DEHP) in PVC medical devices: a critical review. Am J Ind Med 2001;39:100–11. 11148020 10.1002/1097-0274(200101)39:1<100::aid-ajim10>3.0.co;2-q

[16] Yang HW Luan SF Zhao J Improving hemocompatibility of styrene-b-(ethylene-co-butylene)-b-styrene elastomer via *N*-vinyl pyrrolidone-assisted grafting of poly(ethylene glycol) methacrylate. Polymer 2012;53:1675–83.

[17] Yang HW Luan SF Zhao J *N*-vinyl pyrrolidone-assisted free radical functionalization of glycidyl methacrylate onto styrene-b-(ethylene-co-butylene)-b-styrene. React Funct Polym 2010;70:961–6.

[18] Luan SF Zhao J Yang HW Surface modification of poly(styrene-b-(ethylene-co-butylene)-b-styrene) elastomer via UV-induced graft polymerization of *N*-vinyl pyrrolidone. Colloid Surface B 2012;93:127–34. 10.1016/j.colsurfb.2011.12.027 22264686

[19] Luan S Yin J Li Z Resin compound containing a functionalized polypropylene and a functionalized styrenic thermoplastic elastomer. U.S. Patent 8119702, 2012.

[20] Mendes GCC Brandao TRS Silva CLM. Ethylene oxide sterilization of medical devices: a review. Am J Infect Control 2007;35:574–81. 17980234 10.1016/j.ajic.2006.10.014

[21] Rutala WA Weber DJ. Infection control: the role of disinfection and sterilization. J Hosp Infect 1999;43:S43–55. 10658758 10.1016/s0195-6701(99)90065-8

[22] Lucas AD Merritt K Hitchins VM Residual ethylene oxide in medical devices and device material. J Biomed Mater Res B 2003;66B:548–52. 10.1002/jbm.b.10036 12861606

[23] Yahata K Fujishiro K Hori H An investigation of symptoms in ethylene oxide sterilization workers in hospitals. J Occup Health 2001;43:180–4.

[24] Clough RL. High-energy radiation and polymers: a review of commercial processes and emerging applications. Nucl Instrum Methods B 2001;185:8–33.

[25] Steller R Zuchowska D Meissner W Crystalline structure of polypropylene in blends with thermoplastic elastomers after electron beam irradiation. Radiat Phys Chem 2006;75:259–67.

[26] Luan SF Shia H Yao ZH Effect of electron beam irradiation sterilization on the biomedical poly (octene-co-ethylene)/polypropylene films. Nucl Instrum Methods B 2010;268:1474–7.

[27] Luan SF Yang HW Shi HC Stabilization of polypropylene, polypropylene blends with poly (styrene-b-(ethylene-co-butylene)-b-styrene) under irradiation: a comparative investigation. Nucl Instrum Methods B 2011;269:94–9.

[28] Alariqi SAS Kumar AP Rao BSM Stabilization of gamma-sterilized biomedical polyolefins by synergistic mixtures of oligomeric stabilizers. Polym Degrad Stab 2006;91:2451–64.

[29] Alariqi SAS Kumar AP Rao BSM Stabilization of gamma-sterilized biomedical polyolefins by synergistic mixtures of oligomeric stabilizers. Part II. Polypropylene matrix. Polym Degrad Stab 2007;92:299–309.

[30] Cai CH Shi Q Li LL Grafting acrylic acid onto polypropylene by reactive extrusion with pre-irradiated PP as initiator. Radiat Phys Chem 2008;77:370–2.

[31] Shi HC Shi DA Yin LG Preparation of PP-g-PEG by using partial pre-irradiated polypropylene as initiator and its properties. Polym Bull 2010;65:929–40.

